# Diagnosis of Anxiety in COPD Patients: Usefulness of the HADS Test in Primary Care and Pulmonology Services

**DOI:** 10.3390/jpm14070713

**Published:** 2024-07-02

**Authors:** Enrique Barrueco, Miguel A. Hernández-Mezquita, Vanesa Hidalgo-Sierra, Rosa Cordovilla, Javier Olivera-Pueyo, Javier Galán

**Affiliations:** 1 Ciudad Rodrigo Primary Health Care Center, Health Service Castilla-León (SACyL), Ciudad Rodrigo, 37500 Salamanca, Spain; enbarrueco@saludcastillayleon.es; 2 Institute of Biomedical Research of Salamanca (IBSAL), 37007 Salamanca, Spain; rcordovilla@saludcastillayleon.es; 3 Department of Pulmonology, Salamanca University Hospital, Health Service Castilla-León (SACyL), 37007 Salamanca, Spain; 4 Department of Medicine, Salamanca University, 37007 Salamanca, Spain; 5 Tejares Primary Health Care Center, Health Service Castilla y León (SACyL), 37008 Salamanca, Spain; vhidalgos@saludcastillayleon.es; 6 Department of Psychiatry, Huesca University Hospital, Aragón Health Service (SAS), 22004 Huesca, Spain; joliverap@aragon.es; 7 Faculty of Health and Sports Sciences, Zaragoza University, 22004 Huesca, Spain; 8 Department of Internal Medicine, Salamanca University Hospital, Health Service Castilla-León (SACyL), 37007 Salamanca, Spain; jagalanj@saludcatillayleon.es

**Keywords:** COPD, anxiety, prevalence, risk factors

## Abstract

Anxiety disorders, characterized by excessive fear and anxiety, are increasingly recognized as significant comorbidities in chronic diseases such as chronic obstructive pulmonary disease (COPD). This study aimed to evaluate the prevalence of anxiety in COPD patients referred from primary care centers to pulmonology services and to identify predictive factors for anxiety. This was a multicentric, observational, and prospective study in which 293 COPD patients were recruited, and they underwent comprehensive respiratory and smoking histories, spirometry, and anxiety assessments using the Hospital Anxiety and Depression Scale (HADS). The results showed a diagnosis of suspected anxiety in 85 patients (29.0%): 17 possible and 68 with a strong suspicion. The study found significant associations between anxiety and factors such as gender (women had a risk that was 3.5 times higher than men), weight, and body mass index (BMI). Disease severity, smoking status, and clinical manifestations did not significantly influence anxiety prevalence. These findings underscore the need for systematic psychological evaluations in COPD management and support the use of simple diagnostic tools like the HADS to facilitate referrals to mental health services. Addressing anxiety in COPD patients could potentially improve their quality of life and disease outcomes. This study highlights the importance of a multidisciplinary approach involving family medicine, pulmonology, and psychiatry to optimize COPD patient care and suggests that future research should focus on the impact of anxiety treatment on COPD progression. These insights call for integrating psychological assessments into routine clinical practice for comprehensive COPD management. The registration number is 10.14201/gredos.148549.

## 1. Introduction

Anxiety disorders are an emerging pathology characterized by excessive fear and anxiety along with related behavioral problems that are severe enough to cause significant distress or impairment in personal, family, and social [1], functioning of those who suffer from them. Recently, anxiety has begun to be recognized as a comorbidity that affects the evolutionary course of other diseases, such as chronic obstructive pulmonary disease (COPD), in which it can significantly alter the clinical expression of the disease. The overlap between high levels of comorbidity and COPD is associated with a poor clinical and prognostic outcome [2].

The prevalence of anxiety in the adult population ranges from 6.7% to 13% [3], and is 9.2% in women [4]. The anxiety prevalence in patients with COPD is highly variable, ranging from 6% to 70%, depending on the type of population [5], the severity of the disease [6], and the instruments used for diagnosis, but it is higher in COPD patients than in the healthy population; various authors have also reported an anxiety prevalence in COPD patients of around 44% [7,8].

A patient with COPD has a 2.5 times higher risk of experiencing anxiety than the general population [9,10]. In a systematic review of 152 articles published in the Revue des Maladies Respiratoires, Underner et al. [11] found an anxiety prevalence ranging from 6.7% to 58% in COPD patients and observed positive associations between anxiety prevalence and the risk of respiratory exacerbations. Another systematic review by Pooler and Beech [12], observed an increased risk of exacerbations, longer hospital stays, and a higher risk of mortality.

Perpiña-Galvan [13], points out that “many researchers believe that a systematic assessment of possible psychological disorders such as anxiety and depression should be part of the management of respiratory disease. Despite the evident relationship between anxiety and respiratory disease, anxiety disorders remain underdiagnosed and undertreated in respiratory patients”.

Despite the evidence showing a high prevalence and an exceedingly negative impact of depression and anxiety in patients with COPD, depression and anxiety are rarely screened in clinical practice. Possible explanations for these findings are that depression and anxiety are not routinely screened in medicine clinics, or that the patients may not report their depression and anxiety symptoms to their doctors [14]. Given these facts, the aim of this study was to evaluate the suspected diagnosis of anxiety in patients with COPD referred from primary care centers to pulmonology services, using a simple test such as the HADS test, prior to their possible referral to specialized mental health services.

The secondary objective was to assess the existence of predictive factors that could alert the doctors responsible for the diagnosis and monitoring of COPD to the presence of a mental disorder such as anxiety, which can influence the disease’s course.

## 2. Materials and Methods

The study protocol has been previously published in another study on depression and COPD [15].

### 2.1. Ethical Aspects

This study has been approved by the Clinical Research Ethics Committee (CEIC) of the Health Area of Salamanca (Código 2020-03-455). All participants signed an informed consent form prior to the study in accordance with the Declaration of Helsinki and World Health Organization standards for observational studies [16]. During the development of this study, no alteration was required in the medical procedures for which the patients attended medical consultations. Patient data were treated confidentially in accordance with the provisions of current legislation on personal data protection and the conditions outlined by Act 14/2007 on biomedical research [17]. The patients were informed of the proposed objectives and benefits of the project, and they were free to withdraw from the study at any time.

### 2.2. Patients and Study Design

The patients recruited for the study were referred from two primary care centers to their reference pulmonology services, which are departments that share care for COPD patients. For the diagnosis and classification of COPD, the recommendations of the GesEPOC [18], and GOLD [19], guidelines that took effect in 2022 were followed.

This was an observational, multicentric, prospective, transversal study with a non-probabilistic sample. All patients were observed during a consecutive period of six months, regardless of age, sex, stage of their COPD, or treatment.

Patients who agreed to participate underwent a complete respiratory history, an updated smoking history, and spirometry with a bronchodilator test to verify that they met the diagnostic criteria for COPD. The diagnosis of anxiety was made using the corresponding scale of the HADS [20] test based on the Spanish validation [21], and previous studies carried out with said scale [22]. The cutoff point for establishing a suspicion of anxiety was set at a value greater than 7.

### 2.3. Statistical Analysis

For the descriptive analysis, absolute and relative frequencies of all qualitative variables and the mean and standard deviation for quantitative variables were determined. A comparison of scores was performed using the student’s *t*-test for independent samples.

An analysis of factors influencing the psychiatric diagnosis was conducted using binary logistic regression. In each situation, to estimate the risk in a univariate manner, the odds ratio (OR), with its corresponding 95% confidence intervals, was used. Finally, for the evaluation of predictors of anxiety, a multivariate analysis was conducted using logistic regression, including the significant variables from the binary analysis and another multivariate analysis of all the variables included in the study.

The criterion for significance was set at *p* < 0.05. All statistical analyses were performed using IBM SPSS Statistics software version 20 (International Business Machines, Armonk, NY, USA) under the supervision of the company Cenit Support Systems S.L.U. (http://www.cenitss.es/, accessed on 15 January 2024) on the business campus of the University of Salamanca.

## 3. Results

The study included 229 men and 64 women with a mean age of 68.2 ± 10.3 years (range 40 to 91 years). The mean height was 165.8 ± 8.6 cm (range 140 to 191 cm), the mean weight was 74.4 ± 15.4 kg (range 38 to 165 kg), and the mean body mass index was 27 (range 18 to 45). A total of 127 patients live in rural areas and 166 in urban areas; 47 patients (19.5%) live alone.

At the time of inclusion in the study, 93 patients (31.7%) still smoked and 200 (68.3%) did not, although all had been smokers with an average consumption of 25.5 ± 13.6 cigarettes/day, an average smoking history of 39.7 ± 11.5 years, which corresponds to a pack-year index (PYI) of 50.7 ± 29.9. For the ex-smokers, the mean number of years since they quit smoking was 11.7 ± 0.7.

The mean time from COPD diagnosis to inclusion in the study was 6.6 ± 6.4 years. The predominant symptom was exertional dyspnea grades II and III according to the mMRC (modified Medical Research Council) scale (29.7% and 18.9%, respectively); the mean BODEx index value was 2.93 ± 1.99. In the past year, 52.6% of the patients had experienced an exacerbation (mean of 2 ± 1.4).

Regarding the severity of airway obstruction, according with GOLD Guidelines, 127 patients (43.4%) had moderate obstruction, and 98 patients (33.45%) had severe obstruction. Another 42 patients (14.33%) had mild obstruction, and 26 (8.87%) had very severe obstruction.

According to the criteria of the Spanish COPD Guidelines (GesEPOC) [17], 64.5% of the patients were classified as high risk and 35.5% as low risk; the most frequent phenotype was the exacerbator with emphysema (109 patients; 37.2%) and the least frequent was the mixed phenotype (20 patients; 6.8%).

Based on the classification of the Global Initiative for Chronic Obstructive Lung Disease (GOLD), which is valid until 2022 [18], a distribution of patients across different groups was observed. The most frequent groups (B and D) consisted of 169 patients (57.7%), and these groups included patients with more symptoms. The less frequent groups (A and C) consisted of 124 patients (42.3%), and these groups included patients with fewer symptoms.

The average score on the COPD Assessment Test (CAT) was 14.6 ± 6.9 points. The clinical impact was analyzed according to the same scale used by Jiménez et al. [23]. Thus, 83 patients (28.3%) reported no impact on their quality of life or a very low impact (CAT: ≤10), and 210 patients (71.7%) reported some impact. Of these, 162 (55.3%) reported a moderate impact (CAT: 11–20), 40 (13.6%) reported a high impact (CAT: 21–30), and 8 (2.73%) reported a very high impact (CAT: 31–40).

An assessment of anxiety was conducted using the HADS test. Table 1 summarizes the results of the Hospital Anxiety and Depression Scale (HADS) for anxiety. The participants were divided into three groups based on their scores: 7 or less, between 8 and 10, and 11 or more. The table shows the frequency and percentage of participants in each group, along with the mean score and the *p*-value for each group. The *p*-value indicates statistical significance, with all values being <0.001.

Table 1 presents the observed results on the anxiety scale of the HADS test. The cutoff point for establishing the suspicion of anxiety was set at a value > 7. Frequency analysis showed 208 patients without anxiety. A diagnosis of suspected anxiety was established in 85 patients (29.0%): 17 possible and 68 with a strong suspicion.

Out of the 293 patients, 27 (9.2%) had a prior clinical diagnosis of anxiety established by a mental health unit, while 266 did not. Table 2 shows the average scores in each of the three groups studied. Despite the large difference in the number of patients with a prior diagnosis compared to the other two groups, the observed differences were significant. The average score was 4.9 points higher for patients with a prior diagnosis of anxiety compared to the undiagnosed group, confirming the usefulness of the test used.

Bivariate and multivariate analyses were conducted to determine the influence of the variables included in the study on the likelihood of patients suffering anxiety. Table 3 summarizes the results of the logistic regression analysis for predictors of anxiety. It includes the Wald statistic, *p*-value, odds ratio (OR), and the 95% confidence interval (CI) for each variable.

Only personal factors such as being female, weight, and BMI showed significant influence. The Wald test indicates that being female had a positive influence (higher probability), while weight and BMI had an inverse influence, meaning that lower weight was associated with a higher probability of experiencing anxiety. The risk analysis showed that women were 3.5 times more likely to experience anxiety than men. Regarding weight, the value was less than 1, indicating that for each kilogram reduction in weight, the risk of experiencing anxiety increased by one point (1/0.955 = 1.047), and the same applied to BMI (1/0.907 = 1.102). The influence of these last two variables disappeared in the multivariate analysis, with only being female remaining as a predictive factor.

An analysis of the results showed that personal variables such as age and form of residence (urban or rural) did not have a significant impact. Similarly, variables related to tobacco consumption, clinical manifestations, lung function, and disease classification according to GesEPOC (both phenotype and risk) or GOLD did not show a significant influence. Although a trend was observed in some cases, it was not strong enough to be considered influential.

## 4. Discussion

A definitive diagnosis of a mental disorder is clinical and performed by psychiatrists in mental health units through a structured psychiatric interview. However, there are a wide range of tests available, including the HADS, which facilitates the establishment of a presumptive diagnosis and allows for the referral of the patient to a psychiatrist for a definitive diagnosis and subsequent joint follow-up. In a previous study by González et al. [24], a high concordance was found between a positive HADS (using the same cutoff points as in our study) and a confirmatory psychiatric diagnosis of anxiety (Cohen’s Kappa index of 0.885, *p* < 0.001). Therefore, due to its simplicity, the HADS test may be useful for clinicians who are not experts in diagnosing psychosocial disorders.

Mental disorders are more prevalent in patients with COPD than in the general population and are more prevalent in women than in men [25]. Anxiety and depression are underdiagnosed in this group of patients, and anxiety is possibly even more underdiagnosed than depression.

Brenes indicated in Psychosom. Med. in 2003 [10] that anxiety was three times more common in patients with COPD than in the general population. However, as we have already noted, the reported prevalence is highly variable: 5.6% in the study by Kull et al. [26], 26.5% in the study by González-Gutiérrez et al. [27], and 8.1% in the most recent study by Xiao et al. [28]. The prevalence observed in our study, using the HADS test and setting the scale cutoff point at ≥ 8, was 28.9%, with a total of 85 patients scoring eight points or higher. This is close to the prevalence found by González-Gutiérrez et al. [24], which had a population group similar to ours [15]. It is noteworthy that in our study, only 27 patients (9.2%) had an established anxiety diagnosis, leading to the conclusion that there was significant underdiagnosis in the studied population. The EPISCAN II study [29], a population-based study that also used the HADS test, showed an anxiety prevalence of 27.4% in the COPD group.

When studying the psychiatric comorbidities that influence COPD, depression is more commonly considered than anxiety. The opposite is true for asthma, possibly due to a gender bias resulting from a higher prevalence of asthma in women and a higher prevalence of COPD in men. However, anxiety is a common comorbidity in COPD and has a significant impact on the quality of life and survival of these patients. In fact, the COTE index (COPD Specific Comorbidity Test) includes anxiety among the specific comorbidities associated with COPD that determine increases in mortality [30].

An important aspect is to analyze the existence of possible predictive factors that could serve as warning signs for clinicians who suspect the possibility of anxiety. In this regard, only a few personal factors such as being female, weight, and BMI showed significance, while other personal factors such as age, which had shown significance in the study by González et al. [24], did not show a relationship in our study. Regarding disease-related factors, they did not show a significant influence, although there was a trend related to a greater severity of the disease, which makes early identification of anxiety in these patients more challenging.

These results contrast with those observed by our research group in relation to depression [15], in which the personal factors that showed influence included age and living alone. Additionally, other aspects related to the disease itself, such as the CAT score, BODEx index, degree of bronchial obstruction, phenotype, and risk factors (GesEPOC), as well as GOLD classification grades C and D, also showed significance.

Anxiety is more prevalent in women, while COPD is more prevalent in men, leading to few women being included in studies on patients diagnosed with COPD. Mayoral et al. [31] observed that among women diagnosed with COPD, 43.1% had a history of anxiety. In our study, only 21.8% of the participants were women; however, this is a higher percentage than that of other studies. The fact that being female is consistently reaffirmed as a predictive factor in all analyses conducted gives greater significance to this finding.

Smoking cessation and pulmonary rehabilitation have been shown to be the only factors capable of modifying the evolutionary course of COPD. Bronchodilator treatment improves the quality of life for these patients and reduces their perception of dyspnea. However, in the case of patients with comorbid anxiety, treating the anxiety could improve their perception of dyspnea in particular and contribute to an overall improvement in quality of life. Despite this, less than one-third of patients with COPD receive adequate treatment for anxiety. This aspect was not addressed in our study, but it is one of the factors to be considered in future research.

This study has limitations derived from the specific Spanish population that was included and the correlational nature of the study. These limitations could affect the generalizability of the study’s findings, especially regarding the predictive factors.

The most updated versions of COPD management guidelines, such as GesEPOC [18] and GOLD [32], recommend a specific assessment of the psychosocial status of these patients. However, these recommendations are far from routine clinical practice, likely due to multifactorial causes, including diagnostic difficulty. Therefore, it is of interest to have simple tests such as the HADS test that can guide the presumptive diagnosis of anxiety and allow for the referral of patients to mental health units.

## 5. Conclusions

Anxiety is a prevalent mental illness in patients with COPD, but it is underdiagnosed. The use of simple tools like the HADS test in primary care and pulmonology services could facilitate a diagnosis and the referral of patients to mental health units. A multidisciplinary approach involving family medicine, pulmonology, and psychiatry would be a strategic alliance that could help improve the health status of COPD patients and the overall course of the disease.

## Figures and Tables

**Table 1:** HADS anxiety scale results.

Score Range	Frequency	Percentage	Mean Score	*p*-Value
≤7	208	70.98	2.76 ± 2.3	<0.001
8–10	17	5.74	8.35 ± 0.5	<0.001
≥11	68	23.2	12.92 ± 2.3	<0.001

**Table 2:** Mean scores by group on the anxiety scale using the HADS test.

Group	Frequency	Percentage	Mean Score	*p*-Value
Global Population	293	100	5.45 ± 4.8	
Without Prior Anxiety Diagnosis	266	90.8	4.20 ± 4.18	*p* < 0.001
With Prior Anxiety Diagnosis	27	9.2	9.18 ± 4.66	*p* < 0.001

**Table 3:** Variables that showed influence in the bivariate analyses.

Variable	Wald	*p*-Value	OR	95% CI
Male	8.393	0.004 *	0.401	0.232–0.695
Female	2.897	0.004 *	3.518	1.502–8.241
Weight	−2.840	0.005 *	0.955	0.926–0.986
BMI	−2.151	0.031 *	0.907	0.829–0.991

* *p* < 0.05.

## Data Availability

The data presented in this study are available on request from the corresponding author and in the Gredos Document Repository (http://hdl.handle.net/10366/148549, accessed on 24 April 2024).

## References

[1] (2023). International Statistical Classification of Diseases and Related Health Problems.

[2] Di Raimondo D., Pirera E., Pintus C., De Rosa R., Profita M., Musiari G., Siscaro G., Tuttolomondo A. (2023). The Role of the Cumulative Illness Rating Scale (CIRS) in Estimating the Impact of Comorbidities on Chronic Obstructive Pulmonary Disease (COPD) Outcomes: A Pilot Study of the MACH (Multidimensional Approach for COPD and High Complexity) Study. J. Pers. Med..

[3] Albarracin G., Rovira J., Carreras L., Rojas J. (2008). Aspectos económicos y epidemiológicosde los trastornos de ansiedad generalizada: Una revisión de la literatura. Actas Esp. Psiquiatr..

[4] Ministerio de Sanidad, Consumo y Bienestar Social (2019). Encuesta Nacional de Salud (ENSE), España 2017.

[5] López-García F., Pineda-Cuenca M., Custardoy-Olavarrieta J. (2007). Ansiedad y depresión en la EPOC. Rev. Clin. Esp..

[6] Usmani Z.A., Carson K.V., Heslop K., Esterman A.J., De Soyza A., Smith B.J. (2017). Psychological therapies for the treatment of anxiety disorders in chronic obstructive pulmonary disease. Cochrane Database Syst. Rev..

[7] Fernández-Villar A., Fernández-García S., Represas-Represas C. (2020). El componente social de la enfermedad pulmonar obstructiva crónica: ¿un rasgo tratable de la enfermedad?. Arch. Bronceumol..

[8] Fernández-García S., Represas-Represas C., Ruano-Raviña A., Mosteiro-Añón M., Mouronte-Roibas C., Fernández-Villar A. (2020). Perfil social de los pacientes que ingresan por una agudización de EPOC. Un análisis desde una perspectiva de género. Arch. Bronconeumol..

[9] Working group of the GesEPOC (2017). Comorbilidades en la EPOC. Arch. Bronconeumol..

[10] Brenes G.A. (2003). Ansiety and chronic obstructive pulmonary disease: Prevalence, impact and treatment. Psichosom. Med..

[11] Underner M., Cuvelier A., Peiffer G., Perriot J., Jaafari N. (2018). The influence of anxiety and depression on COPD exacerbations. Rev. Des Mal. Respir..

[12] Alison Pooler A., Beech R. (2014). Examining the relationship between anxiety and depression and exacerbations of COPD which result in hospital admission: A systematic review. Int. J. Chron. Obstruct. Pulmon. Dis..

[13] Perpiñá-Galván J., Richart-Martínez M., María José Cabañero-Martínez M.J. (2011). Arch Fiabilidad y validez de una versión corta de la escala de medida de la ansiedad STAI en pacientes respiratorios. Arch. Bronconeumol..

[14] Wu D.W., Chang L.H., Yang P.C., Kuo T.Y., Tsai D.L., Chen H.C., Yuan H.L., Chen P.S., Chen S.C., Lin I.M. (2022). Anxiety Is a Mediator between Heart Rate Variability and Quality of Life in Chronic Obstructive Pulmonary Disease. J. Pers. Med..

[15] Barrueco-Otero E., Refoyo Matellán B., Martín Puente J., Viñado Mañes C., León Subias E., Olivera Pueyo J., Sancho Sanchez C. (2022). Prevalence of Depressive Symptoms, Predictive Factors, and Diagnosis of Suspicion of Depression in Patients with COPD. Aten. Primaria.

[16] World Medical Association (2017). WMA—The World Medical Association-Declaración de Helsinki de la AMM—Principios éticos Para las Investigaciones Médicas en Seres Humanos. https://www.wma.net/es/policies-post/declaracion-de-helsinki-de-la-amm-principios-eticos-para-las-investigaciones-medicas-en-seres-humanos/.

[17] (2007). BOE Ley 14/2007, de 3 de Julio, de Investigación Biomédica. https://www.boe.es/eli/es/l/2007/07/03/14.

[18] Miravitlles M., Calle M., Molina J., Almagro P., Gómez J.T., Trigueros J.A., Cosío B.G., Casanova C., López-Campos J.L., Riesco J.A. (2022). Actualización 2021 de la Guía Española de la EPOC. (GesEPOC). Arch. Bronconeumol..

[19] Global Initiative for Chronic Obstructive Lung Disease (GOLD) (2021). Global Strategy for the Diagnosis, Management, and Prevention of Chronic Obstructive Pulmonary Disease: 2022 Report. https://goldcopd.org/2022-gold-reports/.

[20] Zigmond A.S., Snaith R.P. (1983). The Hospital Anxiety and Depression Scale. Acta Psychiatr. Scand..

[21] Terol-Cantero M.C., López-Roig S., Rodríguez Marín J., Martin Aragón M., Pasto M.A., Reig M.T. (2007). Propiedades psicométricas de la Escala Hospitalaria de Ansiedad y Estrés (HAD) en población española. Ansiedad Estres.

[22] Terol-Cantero M.C., Cabrera-Perona V., Martín-Aragón M.T. (2015). Revisión de estudios de la Escala de Ansiedad y Depresión Hospitalaria (HAD) en muestras españolas. An. Psicol..

[23] Jiménez-Ruiz C., Pascual Lledó F.J., Cícero Guerrero A., Cristóbal Fernández M., Mayayo Uribarri M., Villar Laguna C. (2018). Análisis de la calidad de vida en pacientes con enfermedad pulmonar obstructiva crónica (EPOC) que dejan de fumar. Semergen.

[24] González Gutiérrez M.V., Guerrero Velázquez J., Sánchez Martínez J.A., Casas Maldonado F., González Vargas F. (2014). Prevalencia de ansiedad y depresión en pacientes con EPOC calculada mediante diagnóstico psiquiátrico. Rev. Esp. Patol. Torac..

[25] Laurin C., Lavoie K.L., Bacon S.L., Dupuis G., Lacoste G., Cartier A., Labrecque M. (2007). Sex differences in the prevalence of psychiatric disorders and psychological distress in patients with COPD. Chest.

[26] Kühl K., Schürmann W., Rief W. (2008). Mental disorders and quality of life in COPD patients and their spouses. Int. J. Chron. Obstruct. Pulmon. Dis..

[27] González-Gutiérrez M.V., Guerrero Velázquez J., Morales García C., Casas Maldonado F., Gómez Jiménez F.J., González Vargas F. (2016). Modelo predictivo de ansiedad y depresión en pacientes españoles con enfermedad pulmonar obstructiva crónica estable. Arch. Bronconeumol..

[28] Xiao T., Qiu H., Chen Y., Zhou X., Wu K., Ruan X., Wang N., Fu C. (2018). Prevalence of anxiety and depression symptoms and their associated factors in mild COPD patients from community settings, Shanghai, China: A cross-sectional study. BMC Psychiatry.

[29] Soriano J.B., Alfageme I., Miratvilles M., De Lucas P., Soler-Cataluña J.J., García-Rio F., Casanova C., Gonzalez-Moro J.M., Cosío B.G., Sanchez G. (2021). Prevalence and Determinants of COPD in Spain: EPISCAN II. Arch. Bronconeumol..

[30] Divo M., Cote C., Torres J.P., Casanova C., Marin J.M., Pinto-Plata V., Zulueta J., Cabrera C., Zagaceta J., Hunninghake G. (2012). Comorbidities and Risk of Mortality in Patients with Chronic Obstructive Pulmonary Disease. Am. J. Respir. Crit. Care Med..

[31] Mayoral S., Díaz Lobato S., Antón E., Ribera X., Unzueta I., Martin A. (2016). Características clínicas y sociodemográficas de mujeres diagnosticadas de Enfermedad Pulmonar Obstructiva Crónica (EPOC) en España: Estudio ECME. Rev. Patol. Respir..

[32] Global Initiative for Chronic Obstructive Lung Disease (GOLD) (2023). Global Strategy for the Diagnosis, Management, and Prevention of Chronic Obstructive Pulmonary Disease: 2024 Report. https://goldcopd.org/wp-content/uploads/2023/11/KEY-CHANGES-GOLD-2024.pdf.

